# Reflections on the Passing Year

**Published:** 1890-12

**Authors:** 


### Editorial.

### REFLECTIONS ON THE PASSING YEAR.

The close of the year's work leads naturally to the thought, has there been any material gain in the dental profession during the period, and, if any, in what direction? A year without advancement is a year lost. A year of stagnation is disastrous to the best interests of any calling, as it means a double effort to bring up the lost months and a universal crippling of energy.

It is questionable whether any year can be placed in this category. Insensibly there is growth along mental and physical lines, while the extrinsic evidences may be lacking to demonstrate it.

The last twelve months have not shown any marked advance; indeed, the pessimist may say, in sorrow, the present days are not those of the past. Verbiage has given place to solid work, demonstration to theory, a want of familiarity with the methods and discoveries of a past generation and a persistent attempt to destroy this work that cost so much to make the present more brilliant by contrast. These are familiar expressions to readers, and when the cry is heard, "Our conventions are no longer of value in a scientific sense; our local associations are lacking in life and have ceased to be more than social reunions," we are forced to ask, is this true? It is unquestionably the fact that there is sufficient evidence to sustain the charge; but the caviller is, unfortunately, the one least able or willing to look at professional life philosophically.

The past year has not been a weakling among the years. It is true that, while it has seen the publication of more works of extraordinary merit than any previous decade has furnished, the work has not been done this year, nor has the twelve months been prolific in great results, judged from a scientific standard. There must be periods of rest, periods in which large bodies of men seem to stagnate; but it is simply the inertia of mental force, and will, in the advancing time, give a momentum doubly aggregating any temporary loss.

The fact that there has been criticism along all these branches of professional work is an evidence in itself that there is mental activity and unrest, and that means, in the near future, change and progress.

The year just about closing has not been a lost year. It has witnessed in many directions a healthy growth. This has been clearly shown to the intelligent observer in all our conventions, whether local, State, or national. It has been a year of conventions. They have demonstrated, if nothing else, that the profession is actively interested in these means of training, and regards them as important auxiliaries to the schools. The latter have not been laggards, and have used the passing months in perfecting the advanced work necessary in the increased length of course before them. There has been exhibited an impatience with old methods that indicates health, even though this activity may not lead to great results. The papers and discussions in all the societies have shown dissatisfaction with existing modes. Theory and practice have come in conflict. Modern methods in preventing caries have had to meet the advocates of the still older processes and re-prove themselves. Methods supposed to have been settled show signs of weakness. There is nothing, apparently, too sacred in the list of dental operations to be shielded from attack. This is as it should be. The truly progressive mind welcomes all criticism. It is the open door to knowledge, and without it science would cease its labors, progress would stop, and man himself would lapse into barbarism.

The year just opening promises to be active in many things. It will undoubtedly be one of change, of mental growth. As the dental profession is a part of the great thinking world, it should do its part to stir the intellectual current as never before, and every man who claims to be a member should resolve to be one in the active body, attend the conventions, and by his presence help to make a solid phalanx ever advancing towards the goal of professional unity.

